# Barriers and opportunities for implementation of a brief psychological intervention for post-ICU mental distress in the primary care setting – results from a qualitative sub-study of the PICTURE trial

**DOI:** 10.1186/s12875-023-02046-0

**Published:** 2023-05-06

**Authors:** Linda Sanftenberg, Antina Beutel, Chris Maria Friemel, Robert Philipp Kosilek, Maggie Schauer, Thomas Elbert, Ulf-Dietrich Reips, Sabine Gehrke-Beck, Tomke Schubert, Konrad Schmidt, Jochen Gensichen, for the PICTURE-study team, Christine Adrion, Matthias Angstwurm, Antje Bergmann, Gerhard Bielmeier, Andrea Bischhoff, Ralph Bogdanski, Franz Brettner, Christian Brettschneider, Josef Briegel, Martin Bürkle, Johanna Dohmann, Peter Falkai, Thomas Felbinger, Richard Fisch, Hans Förstl, Benjamin Fohr, Martin Franz, Patrick Friederich, Chris Maria Friemel, Jürgen Gallinat, Herwig Gerlach, Andreas Güldner, Hanna Hardt, Christoph Heintze, Andreas Heinz, Axel Heller, Christian von Heymann, Petra Hoppmann, Volker Huge, Michael Irlbeck, Ulrich Jaschinski, Dominik Jarczak, Stefanie Joos, Elisabeth Kaiser, Melanie Kerinn, Frank-Rainer Klefisch, Stefan Kluge, Roland Koch, Thea Koch, Michelle Kowalski, Hans-Helmut König, Peter Lackermeier, Karl-Ludwig Laugwitz, Yvonne Lemke, Achim Lies, Klaus Linde, Daniela Lindemann, Dagmar Lühmann, Stephanie May, Ludwig Ney, Jan Oltrogge, Wulf Pankow, Sergi Papiol, Maximilian Ragaller, Nikolaus Rank, Lorenz Reill, Hans-Peter Richter, Reimer Riessen, Grit Ringeis, Ann Rüchhardt, Gustav Schelling, Jörg Schelling, André Scherag, Martin Scherer, Antonius Schneider, Gerhard Schneider, Jürgen Schneider, Julia Schnurr, Susanne Schultz, Thomas G Schulze, Karin Schumacher, Peter Spieth, Franka Thurm, Thomas Vogl, Karen Voigt, Andreas Walther, Dietmar Wassilowsky, Cornelia Wäscher, Steffen Weber-Carstens, Regina Wehrstedt, Roland Weierstall-Pust, Marion Weis, Georg Weiss, Harald Well, Christian Zöllner, Bernhard Zwissler

**Affiliations:** 1 Institute of General Practice and Family Medicine, University Hospital, LMU Munich, Nußbaumstr. 5, 80336 Munich, Germany; 2 Department of Psychology, University of Konstanz, Konstanz, Germany; 3 Charité – Universitätsmedizin Berlin, Institute of General Practice and Family Medicine, Berlin, Germany

**Keywords:** Post-traumatic stress disorder, Post-intensive care syndrome, Narrative exposure therapy, Qualitative analysis, Mental health

## Abstract

**Background:**

The results of critical illness and life-saving invasive measures during intensive care unit treatment can sometimes lead to lasting physical and psychological impairments. A multicentre randomized controlled trial from Germany (PICTURE) aims to test a brief psychological intervention, based on narrative exposure therapy, for post-traumatic stress disorder symptoms following intensive care unit treatment in the primary care setting. A qualitative analysis was conducted to understand feasibility and acceptance of the intervention beyond quantitative analysis of the main outcomes in the primary study.

**Methods:**

Qualitative explorative sub-study of the main PICTURE trial, with eight patients from the intervention group recruited for semi-structured telephone interviews. Transcriptions were analysed according to Mayring's qualitative content analysis. Contents were coded and classified into emerging categories.

**Results:**

The study population was 50% female and male, with a mean age of 60.9 years and transplantation surgery being the most frequent admission diagnosis. Four main factors were identified as conducive towards implementation of a short psychological intervention in a primary care setting: 1) long-term trustful relationship between patient and GP team; 2) intervention applied by a medical doctor; 3) professional emotional distance of the GP team; 4) brevity of the intervention.

**Conclusion:**

The primary setting has certain qualities such as a long-term doctor-patient relationship and low-threshold consultations that offer good opportunities for implementation of a brief psychological intervention for post-intensive care unit impairments. Structured follow-up guidelines for primary care following intensive care unit treatment are needed. Brief general practice-based interventions could be part of a stepped-care approach.

**Trial registration:**

The main trial was registered at the DRKS (German Register of Clinical Trials: DRKS00012589) on 17/10/2017.

**Supplementary Information:**

The online version contains supplementary material available at 10.1186/s12875-023-02046-0.

## Background

Worldwide, the number of patients admitted to an intensive care unit (ICU) is increasing, and improvements in medical technology and treatment helps more patients to survive critical illness [1–3]. Consequently, a growing number of people is discharged from ICU to specialized clinics, rehabilitation facilities and outpatient care [4]. ICU treatment often results in long-term sequelae, resulting in restricted health-related quality of life [5–8]. The post intensive care syndrome (PICS) is a combination of cognitive, mental and physical signs and symptoms, whereas the presentation is very variable [9, 10]. The symptoms can last for a few months to many years post discharge. Mental symptoms include anxious or depressed mood, sleep disturbances, cognitive impairment and post-traumatic stress [11–13].

The risk of developing psychiatric illness as depression, anxiety and post-traumatic stress disorders PTSD is estimated to be up to 62% [14]. However, psychological services available to patients following an ICU treatment are rare [15, 16].

The long-term follow up of patients after ICU treatment is given mainly in primary care. The GP team knows their patients before the ICU treatment started and will support them for years afterwards. Therefore, the PICTURE trial (“PTSD after ICU survival”) has been initiated to improve the follow-up care of ICU patients in primary care by testing a brief talking therapy with case management. The intervention is based on the narrative exposure therapy (NET), a well-established combination of testimony therapy and cognitive-behavioural therapy, originally developed for low-income countries [17]. Assuming a disturbed memory formation for a traumatic event the NET aims to rebuild memory by a detailed and chronological review of the traumatic scenario in which fragmented trauma memories can be embedded. By using targeted questioning the therapist facilitates a vivid narration and mental re-exposure to the traumatic event in the patient. Details are described elsewhere [18]. Furthermore, PICTURE was developed to raise awareness among GP teams and within society towards this topic [18].

In Germany, GP teams provide support and counselling for psychosomatic illnesses and care for people with mental health issues but do not regularly offer psychotherapy or other psychological interventions [19]. Psychological and psychotherapeutical services are often not easily available [20–22] and the stigma associated with mental health care may be a barrier in seeking adequate treatment [23]. The implementation of a PTSD intervention in primary care might overcome this barrier. However, it is unknown, if this approach would be acceptable for patients. Furthermore, the success of an implemented intervention strongly depends on subjective assessment among the involved patients [24]. Therefore, the aim of this qualitative study is to analyse the role of the GP team concerning the practical implementation of the NET assessed by participating patients within the PICTURE trial.

## Methods

### Setting and participants

The PICTURE trial is a German multicentre study, conducted since 2017 at the Institute of General Practice and Family Medicine, Ludwig-Maximilians-University Munich and at the Institute of General Practice and Family Medicine, Charité—Universitätsmedizin Berlin. Adult patients with a total score of at least 20 points on the posttraumatic diagnostic scale (PDS-5) [25] corresponding to moderate symptom severity three months after ICU were equally randomized to two groups: NET combined with case management (intervention) and improved treatment as usual (iTAU, control). NET is delivered by a GP (three sessions each 30 min, S-1 “Lifeline”, S-2 and S-3 narrative exposition to traumatic ICU events, followed by 10 phone contacts to support by the case manager). In more detail, the intervention consists of a first session in which the patient creates a graphical representation of his or her biography using a lifeline (S-1). Afterwards, there are two more sessions in which the patient recounts the stressful situations to recover contextual details of the traumatic event (S-2 and S-3) [17, 18]. The upcoming telephone monitoring by the medical assistant refer to the principles of the chronic care model for special case management, which is focused on proactive patient symptom monitoring [26].

The control group treatment iTAU follows the current German guidelines in PTSD treatment [27]. At six (T1) and 12 month (T2) follow up, the patient-reported outcomes are assessed observer-blinded. All GP teams involved got the information concerning the diagnosis and treatment of PTSD according to current German guidelines. Within the intervention group, the GP teams were additionally trained for at least one hour by a NET specialist (Master of Science, Psychology; CF & AB) [18]. As feasibility and acceptance of a complex intervention in daily practice can be hardly assessed only quantitatively, it is important to apply a mixed-methods evaluation. To analyse the role of the GP team who applies a NET-based intervention, we conducted qualitative studies at both study sites. The qualitative analysis led by the study centre in Munich focused on the patients’ perspective. We applied a phenomenological research approach, as it suits the analysis of the narrative of individual biographical experiences in their complexity [28]. In particular, subjective retrospective assessments that define the success of therapy can be appropriately depicted in individual interviews and can thus be identified according to rules [24]. The reporting follows the reporting guidelines of consolidated criteria for reporting qualitative research) (COREQ) (e-supplement 1) [29]. The study centre in Berlin explored the acceptance of the intervention from the GPs` perspective, these results will be published elsewhere.

### Data collection

Altogether, ten patients from the intervention group of the main trial were invited to the semi-structured interviews via telephone. Two of these invited patients had to be excluded, because of compliance issues (*n* = 1) and timely constraints (*n* = 1).To achieve the greatest possible heterogeneity of the sample, we applied selective sampling to describe different perspectives and expected differences. The representativeness considerations were implemented with regard to sex, age, educational history, place of residence and admission diagnosis. However, extreme variations could not be characterised in our qualitative sub-study, as inclusion and exclusion criteria of the main trial led to a defined study population. Due to our exploratory approach, this study only allows an insight into the patients` perspective, data saturation cannot be assumed.

We conducted semi-structured individual interviews with patients to obtain in-depth information about their perception concerning the role of the GP teams in provision of the combined brief NET with case management. Furthermore, we asked for patient’s perspective on effectiveness of the NET.

All interviews were conducted via telephone from the interviewer`s home office and audiotaped. No one else was present during these interviews besides the participants and researchers. All participating patients already had received the three scheduled NET sessions according to the study protocol of the main trial [18]. An interview protocol was created for each interview to document formalities (interview code, name of the interviewee, date of the interview, contact details) and special occurrences at the initial contact and during the interview. The interviewer was female experienced intensive care nurse (AB; Master of Science, Psychology), who conducted the qualitative study interviews in a self-reflective, neutral manner [30]. Since the interviewer worked on a different intensive care unit than the one from which the patients were invited for the main PICTRUE trial, there was no relationship prior to study commencement. At the beginning of each interview, AB introduced herself as an experienced intensive care nurse and explained that the results of the interviews will be used for her master’s degree and to evaluate some aspects of the NET. None of the interviews had to be repeated. The transcripts were not returned to the participants for comments or correction. There was no feedback of the participants concerning the results or findings.

The interview guideline (e-supplement 2) was created deductively. The theoretical framing was based on published insights concerning posttraumatic stress disorder-related symptoms after critical care [17, 31–36].

In detail, we focused on.

- The aspects of the course and implementation of the NET in primary care are theoretically described in the therapy manual [17]. Questions were focussed on the subjective experience of the patients concerning this implementation in daily practice. Which beneficial and hindering factors are mentioned with regard to the course and implementation of the NET in GP?
- The overall acceptance of the NET intervention. In therapy research, the subjective retrospective assessment of patients is considered to be of great prognostic importance for long-lasting effectiveness within a contextualized environment like the GP setting [24]. Which aspects are mentioned with regard to the acceptance and effectiveness of the NET in the setting of GP?

The interview guideline was discussed in the PICTURE team at both study centres and in qualitative research circles (“Berlin method meeting for qualitative research”). The resulting findings were incorporated.

### Data analysis

The audiotaped interviews were transcribed verbatim with a slight smoothing of the language if necessary (e.g. if dialect was used) [37]. Transcripts were checked for accuracy before they were coded and analysed. The analysis of the collected data was carried out according to Mayring's qualitative content analysis [28] by using MAXQDA ® software (VERBI GmbH, Berlin). Especially with larger amounts of text, this rule-based procedure allows a qualitative evaluation, but also opens up possibilities for quantifying partial aspects. The deductively obtained categories comprise the possibility of forming categories inductively.

The development of the code framework was done independently by two investigators (AB, TS). Created codes were compared and discussed while discrepancies in coding were resolved by consensus. Using the constant comparative method [38], we revised original categories after we compared them with newer categories that emerged in the coding process. Additionally, categories were compared across cases to ensure that they were both representative and inclusive of all cases. We also compared individual patient perspectives regarding the perceived role of the GP in terms of the NET. Both the intra- and intercoder reliability were checked to eliminate any ambiguities in categorizations and thus support the reliability of the analysis. The interviews were conducted from April – June 2021 and lasted 37—70 min.

## Results

Table 1 shows the demographic characteristics of the eight patients who were interviewed. Half of them were female, the mean age was 60.9 years, the majority of the patients had a high level of education and lived in small cities. Reasons for their ICU treatment differed strongly, the most prevalent admission diagnosis was transplantation of lung or liver.

**Table 1:** Patient characteristics (*n* = 8)

**Characteristic**		**Number [n]**
**sex**	male	4
	female	4
**age**	40—50 years	2
	51—60 years	3
	61 -.70 years	3
**educational history**	secondary schools	2
	intermediate maturity	1
	high school diploma	5
**place of residence, number of inhabitants**	< 10.001	3
	10.001 to 25.000	4
	25.001 to 1,5 Mio	1
**admission diagnosis**	cardiac surgery	2
	neurosurgical intervention	1
	polytrauma	1
	sepsis	1
	transplantation (liver or lung)	3

The following presentation of the results is based on the interview guideline (see e-supplement 2). "(…)" means a break in the narrative flow, "[…]" means a shortening of the quote.

Four categories merged from our analysis of patient interviews:

1. Long-term trustful relationship between patient and the GP team

Most patients cited a long-term and trustful relationship to the GP team as major facilitator for the success and acceptance of the intervention. Vice versa, lack of this relationship was identified as a possible barrier.

2) Intervention by a medical doctor

A medical doctor who applies the intervention has the sufficient knowledge about the ICU setting and knowledge of the patient’s medical history. This knowledge was perceived as great advantage in comparison to the more theoretical knowledge attributed to psychotherapists, who are not familiar to these settings. However, a single patient stated that a GP team might be not sufficiently trained to have these kind of psychological conversations and identified the missing qualification as barrier of acceptance.

3) Professional emotional distance of the GP team

The professional emotional distance of the GP team was perceived as relieving for patients and their families in terms of “professional complicity” and was a strong facilitator for acceptance of the intervention.

4) Brevity of the intervention

The brevity of the intervention was mentioned as a barrier for acceptance by two patients. They would have liked more time for the intervention or more than the three sessions offered. Table 2 lists facilitators and barriers cited by the interviewed patients.

1. Long-term trustful relationship between patient and the GP team

**Table 2:** Most frequent cited facilitators and barriers for acceptance of the NET interventions identified by patients

Facilitators identified by patients	Barriers identified by patients
long-term trustful relationship between patient and GP practice staff (*n* = 5)	brevity of the intervention (*n* = 2)
medical doctor applies the intervention (*n* = 4)	unknown GP applies the intervention (*n* = 1)
professional emotional distance of the GP (*n* = 2)	non-psychological expert applies the intervention (*n* = 1)

Five patients cited the long-term trustful relationship between patient and the GP team as the main facilitator for the acceptance of the NET intervention. An interviewee emphasized the importance of home visits as well as the quick and low-threshold accessibility of the GPs practice:

“I won´t hear a word against my doctor (laughs), she is simply the best. How long does it take to find a doctor that makes house calls? Besides that, she is always ready to listen. When I call the practice, then she calls me back within 10 min and that is really super.” (B8; female, 52 years old, secondary school, admission diagnosis: polytrauma)

Other patients also report the very good and trusting relationship with their GP, which they describe as fundamental to the acceptance of the intervention.

"Very positive. (…) Because there is a certain level of trust with her, (…) because that is a prerequisite." (B3; female, 59 years old, high school diploma, admission diagnosis: cardiac surgery)

“The doctor, simply put, knows me..…..inside out, she knows each of my little aches and pains….and that is naturally very helpful, because then I know for sure, that she wouldn´t do anything that is not good for me ….and that´s reassuring.”(B8; female, 52 years old, secondary school, admission diagnosis: polytrauma)

This statement indicates that, from the patient's perspective, mental and physical health are inextricably linked. Good psychological support is therefore assumed, since the long-term doctor-patient relationship also means that the patients` medical history is known.

The telephone contact with the medical assistant was rated neutrally by three respondents, one respondent explicitly emphasized this contact as positive. “I perceived this kind of psychological treatment and the questionnaires quite supportive and then also these calls from the receptionist, who asked me again and again: And, if you look back now, how did you feel in the last two weeks? I think it's good that you just reflect a bit and then maybe classify some things a little different for yourself. Or then maybe you realize why it could have been like that or why you could have reacted like that.” (B5; female, 52 years old, high school diploma, admission diagnosis: sepsis)

Consequently, a female patient identified a primarily unknown GP as a possible barrier for the acceptance of the intervention. She was treated by a previously unknown GP colleague of her GPs practice.

“I think it would be better if a psychotherapist did it, but I would also recommend it if a general practitioner did it. So I think talking about it is definitely better than not doing it at all.” (B5; female, 52 years old, high school diploma, admission diagnosis: sepsis)

2) Intervention provided by a medical doctor

Four interviewed patients assessed the medical background knowledge of their GP team positively.

“It was just good to talk to someone about it and that they were by my side in my illness and that they understand you and how the pills work and how they can work. They were all cool, nice.” (B1; male, 59 years old, secondary schools, admission diagnosis: transplantation (liver or lung))

“Because she also knows this background a bit, on the other hand, of course, had a medical idea and saw my medical course (…).” (B2; male, 66 years old, high school diploma, admission diagnosis: cardiac surgery)

“From her medical point of view, because she's not a psychologist (…) she asks different questions and has a different approach and also this medical understanding, what psychologists, not all, but many do not have (…), […]. It happened on a (…) different level, […] I don't need to explain anything to a medical doctor." (B4; female, 47 years old, intermediate maturity, admission diagnosis: neurosurgical intervention)

Vice versa, another patient resumed, that she would have preferred to get the intervention by a psychotherapist instead of a GP.

"I found […] that it (…) was somehow conducted strictly according to this given pattern.

I: Was that too strict for you?

R: (…) Yes, let's say: I felt it was more like ticking off and less like a […] face-to-face conversation, […] that now it feels to me like a list has been worked through and […] I could imagine that someone with an appropriate training would have approached it a little differently.” (B5; female, 52 years old, high school diploma, admission diagnosis: sepsis)

3) Professional emotional distance of the GP team

Two of the interviewees pronounced the professional distance of the GP team who applied the intervention as very beneficial: "That I can just talk without having to worry that whoever is sitting across from me is concerned about me, but still understands me." B4; female, 47 years old, intermediate maturity, admission diagnosis: neurosurgical intervention).

“… telling that to someone else, someone who is not involved, that just feels good and sheds a little ballast. And when this person does not think that one is nuts, then it is just helpful.”(B8; female, 52 years old, secondary school, admission diagnosis: polytrauma)

This kind of professional knowledge helps some of the interviewees, since they can relieve their families and relatives in particular.

"Well, I notice that my mother is afraid, and […] I try to avoid talking about it as much as possible (…). I don't want that either, I don't want her to be afraid about me, I don't want that (…). And (…) so, that was it with the PICTURE study, I thought it was good, because it's a situation where I could just get rid of things like that, I found it extremely helpful." (B4; female, 47 years old, intermediate maturity, admission diagnosis, admission diagnosis: neurosurgical intervention)

4) Brevity of the intervention

Two patients expressed concerns due to the brevity of the intervention. They would have preferred to have more sessions than three.

“I found that three sessions were a little, I do not want to say, a little short, but four or five would not have been bad. Sometimes I had the wish I may talk about this or that longer.” (B4; female, 47 years old, intermediate maturity, admission diagnosis, admission diagnosis: neurosurgical intervention)

To sum up, we also asked for content-related objective and subjective descriptions of the intervention components. The participants evaluated the first session as profound, but at the same time easy to carry out and valuable to get familiar with this kind of narrative exposition:

“ (…) in the first session she explained the basics, then came at some point kind of (…), how should I name it (…) almost like that family constellation (…), with positive and negative experiences, (…) starting from childhood (…) up to this moment in the intensive care unit.” (B3; female, 59 years old, high school diploma, admission diagnosis: cardiac surgery)

“ (…) the first session with my life up to that point, (…) I think I'm relatively fine with that. (…) but I think I can somehow deal with it a bit and that's why it wasn't difficult” (B2; male, 66 years old, high school diploma, admission diagnosis: cardiac surgery)

“I thought it was really nice, because I liked the idea with the stones and the flowers, (…) looking back, I thought the structure was totally cool and yes, (…) was just good for getting into the topic.” (B4; female, 47 years old, intermediate maturity, admission diagnosis, admission diagnosis: neurosurgical intervention)

The two upcoming sessions were perceived as very helpful to talk about the feelings and impression referring to a traumatic event, like ICU-care.

“ I: And in the other session: Do you remember what that was about?

R: Yes, that was what used to be (…), an earlier event, among other things I remembered, with my father, as a negative event” (B3; female, 59 years old, high school diploma, admission diagnosis: cardiac surgery)”

“To be honest, I can't remember the last one exactly, but the second one, we really did then talk about the event, when I woke up in the night, was tied up and kind of like that was completely helpless and couldn't communicate (...) and no one ever told me what it was like, everything went well or something like that.” (B5; female, 52 years old, high school diploma, admission diagnosis: sepsis)”

“I think it was easier to talk about it, much easier than with the neuropsychologists. With her, talking about that helplessness specifically, I think that's what worries me the most is somehow done, it was much easier with her, with more distance to the events. So then there were no more tears, which, as I said, I certainly did with the neuropsychologists really had too much (…) because somehow I was a bit more distant from it.” (B4; female, 47 years old, intermediate maturity, admission diagnosis, admission diagnosis: neurosurgical intervention)

## Discussion

This explorative qualitative study investigated patient reported factors of the interventions acceptance in eight sociodemographic diverse patients, who received a brief study intervention by their GP based on the NET in the PICTURE trial. We identified four main categories which strongly influence acceptance and perceived effectiveness of the intervention that are associated with the role of the GP: 1) long-term trustful relationship between patient and the GP team 2) medical doctor applies the intervention 3) professional emotional distance of the GP team 4) brevity of the intervention.

Patients suffering from PTSD often show behaviour of avoidance, as well as unspecific symptoms like sleep disturbances, exhaustion, feelings of guilt, depressive symptoms [39]. Therefore, these patients rarely seek psychological support from a psychotherapist or a psychiatrist, although they are severely limited in their everyday functionality and quality of live [40]. Lack of psychological support has important implications for long-term recovery and quality of life following the episode of critical illness [32]. In addition, many of these patients somatise as a consequence of (untreated) psychological complaints [41].

Consequently, patients with PTSD after ICU are treated mainly in general practice. The long-standing trusting relationship provides support and structure, as well as the low-threshold opportunity to address worries and problems. In our study, five out of eight patients addressed the trusting relationship to their GP as supporting factor to face up with their ICU memories and to enter a brief psychological intervention. The diagnostics and the first low-threshold treatment of mental health symptoms should therefore definitely be offered in the setting of GP. This approach has also been established in other areas of mental health [42, 43].

In addition, GPs can use their medical expertise when talking with patients about their ICU stays [44]. For example, side effects of the prescribed drugs, as well as the knowledge of the invasive diagnostic and therapeutic procedures at ICU can be assumed to be known without the patient having to explain them. In addition, not all patients may be able to consciously remember all treatments and procedures [45].

In our study half of the patients interviewed explicitly appreciated the medical expertise of the GP. The medical knowledge of the GP might be especially welcomed for the treatment of medical-related trauma topics (e.g. surgeries, heart attacks, strokes etc.). However, one patient was critical of the less psychotherapeutic counselling techniques of his GP. If the NET would to be integrated in routine primary care, practical exercises and supervision by a NET specialist need to be standardized part of GP.

When the patient is discharged home or to a long-term care facility, patient-centered communication and provision of information again are key to prepare the patient, family, and the primary care team for this next phase of the continuum [43, 46].

The professional emotional distance of the GP team might be very helpful, to talk unconditional about personal emotions, without the additional burden of worrying about a close relative [47]. This is a very important fact, as many patients suffer from feelings of guilt due to the increased attention and family stress, which bring about serious illnesses [48]. The trusted but neutral and professional relationship of GP and patient seems to facilitate also the NET intervention, which is robust and easy to disseminate [49].

Of course, the presented intervention applied by a GP team has not been designed to replace psychotherapy, but to assist those who do not have immediate access to this care. In addition, low-threshold support might not only address the patients themselves, but also relatives and families.

Several limitations of the intervention have been mentioned by the patients. The brevity of the study intervention was explicitly emphasized as negative by two patients. Both respondents were affected by several stressful hospital stays as part of their underlying illness and had comparably very high values in the posttraumatic diagnostic scale (PDS-5) scoring. As they were still suffering from moderate PTSD-symptoms, they do not necessarily require a comprehensive trauma therapy. Severely affected patients, should consider the intervention only as a transitional offer. In such cases, further long-term trauma-focused psychotherapy provided by a specialist might be necessary [32]. However, also severely affected patients may benefit from first positive experiences with NET, which reduces the barrier to seek for psychotherapeutic support.

Patients with mild to moderate PTSD symptoms may recover by NET. It is therefore of the utmost importance to inform outpatient care providers about the clinical signs, symptoms and simple treatment options of mild to moderate PTSD symptoms. Furthermore, they should be informed about somatic indications of a possible stress disorder after ICU.

Timely access to effective treatment is a primary challenge in mental health services. However, when demand exceeds available resources, services may place clients on a waitlist or restrict services [50]. Therefore, our approach tries to use the existing health system to meet the need for mental care. Furthermore, provision of NET within primary care seems to enable easy integration in patient’s daily routine in employment and family. Due to memories related to delusions or feelings of helplessness, loneliness or anxiety, former ICU patients may feel isolated and lonely, even if supported by friends and relatives [46]. Close friends, partners or relatives often have to face the critical health state of the patient and their helplessness [51]. Therefore, psychological distress like anxiety, acute stress disorder, PTSD, depression, and complicated grief symptoms is also observed in partners, close friends and relatives. [8, 13, 52–54] which has been described as (PICS-family) [54]. Therefore, a further development of the NET for caregivers and relatives would be another important building block in the aftercare of this vulnerable patient group in primary care. These aspects of transferability, availability, feasibility and relevance led to an high level of acceptability among the interviewed participants.

### Strengths and limitations of our study

This is the first exploratory study to evaluate barriers and opportunities for the implementation of a brief psychological intervention for post-ICU mental distress in the primary care setting. We gained some valuable insights of the patients` perspective that can be understand as a basis for further examinations.

Although we tried to invite a heterogeneous sample of study participants as possible, a selection bias might be possible. Patients who take part in an evaluation study are usually convinced that the intervention was beneficial for them. It can be assumed, that those patients had a very trustful patient-physician relationship a priori. Furthermore, the GP may be better accepted for dealing with trauma in a medical context than for trauma caused by other means.

In addition, the main trial was tailored for patients suffering from mild to moderate PTSD-symptoms after ICU-care and who have to be open-minded to trauma therapy and narrative exposition therapy. Consequently, it can be assumed that these patients are neither particularly very young nor very old. Since transplant patients wait a long time for their life-saving surgical intervention, these patients are often only affected by a mild to moderate PTSD burden than patients who are affected by other admission diagnoses like sepsis or polytrauma, for example.

Due to our small sample size, data saturation can not be assumed. These results can hardly be transferred to other vulnerable groups such as cognitively impaired/demented patients or children and adolescents. Further studies are needed to examine the transferability of our results to other patient groups, as well as to their caregivers and relatives.

## Conclusion

Clinicians face substantial challenges to deliver evidence-based therapy for treating PICS on a sustainable basis. Diagnostic and therapeutic strategies will not only be needed within the ICU setting but also within primary care settings. This is particularly relevant for posttraumatic symptoms which often occur delayed after ICU discharge. Being well accepted from the patient's perspective, it should be considered how NET can be integrated into standard care. At the same time, it has to be considered how severely affected patients could be referred to specialized settings in a structured manner, in terms of a stepped care approach.

## Supplementary Information

**Additional file 1.**

**Additional file 2.**

## Data Availability

The datasets used and/or analysed during the current study are available from the corresponding author on reasonable request.

## Abbreviations

PICS: Post-ICU syndrome
PTSD: Post-traumatic stress disorder
NET: Narrative exposure therapy
iTAU: Improved treatment as usual
GP: General practice/ general practitioner
ICU: Intensive care unit
