# Supplementary material for: Barriers and opportunities for implementation of a brief psychological intervention for post-ICU mental distress in the primary care setting – results from a qualitative sub-study of the PICTURE trial
Source: BMC Prim Care. 2023 May 6;24:113. doi: 10.1186/s12875-023-02046-0 (PMC10163700; doi:10.1186/s12875-023-02046-0)
Supplement: Supplementary file 2 — Additional file 2. [file 12875_2023_2046_MOESM2_ESM.pdf]

Interview Guideline

**Main question a)**	**Storytelling prompt**	**Definition of the leading question, check: was this mentioned?**	**Concrete Inquiries**
Which facilitating and hindering factors are mentioned with regard to **the course and implementation of NET in primary care**?	Did you feel well informed about the content and process of the therapy at the beginning?<br><br>Please describe to me what talk therapy was like for you. I would like to imagine how it went. Why don't you tell me about it from beginning to end?	Prior knowledge, informedness:<br>Expectations, prior knowledge, motivation?<br><br>Formalities:<br>- Have all sessions taken place? [possibly cross-check with treatment folders].<br>- How much time has passed since completion of NET?<br><br>Content "objective":<br>- What was the content of the sessions? [Address first session separately (psychoeducation and lifeline) and only then the other two].<br>- Was possible amnesia addressed during the sessions?<br><br>Content "subjective."<br>- How was the contact with the primary physician/ medical assistant felt?<br>- Did mental status change during therapy? Possibly worsened?<br>- Experience of re-experiencing? Intrusion?<br>- Effects on everyday life?	Prior knowledge, informedness:<br>- What were your expectations for therapy?<br><br>Formalities:<br>- How many sessions have you had?<br>- When was the last therapy session?<br>- When and where did the conversations take place: At the end of the consultation? In the regular consultation room?<br><br>Content "objective":<br>- How did the session in which you described your life path go?<br>- How did the two sessions go when you recounted your traumatic events?<br>- Was your memory loss/ICU memories addressed during therapy?<br><br>Content "subjective":<br>- How did you feel about the sessions?<br>- How did you feel about the contact with primary physician / medical assistant?<br>- Did you notice any change/improvement/deterioration in yourself during therapy? Did it have an impact on your daily life?<br><br>Enabling and hindering factors<br>- Do you find it easier to talk about the ICU stay with the primary physician than with a therapist*?

		Facilitating and hindering factors:<br>- Is the primary physician offer possibly more low-threshold?<br>- What was perceived positively?<br>- What was perceived negatively?<br>- Suggestions for improvement? Wishes?	- What did you find particularly helpful in the context of the discussions with the doctor/physician (or with the medical assistant, if applicable)?<br>- What did you find difficult?<br>- What did you miss/what else would you have liked?
**Main question b)**	**Storytelling Prompt**	**Definition of leading questions**	**Concrete Inquiries**
Which aspects are mentioned with regard to **acceptance of the NET?**<br>How do patients define success?	What has changed for you since you completed talk therapy? How are you doing today?<br><br>Finally, open-ended question: What do you wish for the future in terms of your mental health?	**Effectiveness of NET or changes since completion of therapy:**<br>- Has there been an overall change in QOL?<br>- What is the course of physical recovery? (Control variable "somatic condition").<br>- What is the course of psychological recovery?<br>- Is the perception of suffering lower?<br>- Question about the social environment<br><br>**Quality of memories:**<br>- What is the quality of memories of the intensive stay? Possibly fewer intrusions? Possibly fewer delusional-distorted memories? Possibly realistic classification?<br>- Dealing with amnesia during therapy: e.g. reconstruction through ICU diary, patient records or similar.<br>- **Acceptance of NET**- Is the therapy perceived as helpful overall helpful.<br>- Subjective assessment of the long-term effectiveness of the therapy	**Effectiveness of NET or changes since completion of therapy:**<br>- How are you doing today in terms of your physical health?<br>- What in particular has contributed to your mental recovery?<br>- What stood in the way of your recovery?<br>- Has your daily life changed since therapy?<br>- Are you supported by those around you?<br><br>**Quality of memories:**<br>- After completing therapy, do you continue to be haunted by distressing memories of the ICU stay?<br>Or if yes: How do you deal with this? What impact does it have on your daily life?<br>- Were you able to reconstruct the period of missing memories through therapy?<br>- Acceptance of NET:<br>- Would you recommend the therapy to others?<br>- What do you wish for your mental health in the future?
